# *Protaetia brevitarsis seulensis* Derived Protein Isolate with Enhanced Osteomodulatory and Antioxidative Property

**DOI:** 10.3390/molecules25246056

**Published:** 2020-12-21

**Authors:** Keya Ganguly, Min-Soo Jeong, Sayan Deb Dutta, Dinesh K. Patel, Seong-Jun Cho, Ki-Taek Lim

**Affiliations:** 1 Department of Biosystems Engineering, College of Agriculture and Life Sciences, Kangwon National University, Chuncheon 24341, Korea; gkeya14@gmail.com (K.G.); sayan91dutta@gmail.com (S.D.D.); dbhu10@gmail.com (D.K.P.); 2 Department of Food Science and Biotechnology, College of Agriculture and Life Sciences, Kangwon National University, Chuncheon 24341, Korea; jminsoo@kangwon.ac.kr

**Keywords:** *Protaetia brevitarsis seulensis*, *Protaetia* protein isolate, biocompatibility, antioxidant, osteogenesis

## Abstract

The osteogenic differentiation of stem cells is profoundly affected by their microenvironmental conditions. The differentiation behavior of stem cells can be tuned by changing the niche environments. The proteins or peptides that are derived by living organisms facilitate the osteogenic differentiation of stem cells. Here, we have evaluated the osteoinductive and antioxidative potential of the *Protaetia brevitarsis seulensis* insect-derived protein for human bone marrow-derived mesenchymal stem cells (hBMSCs). The amino acid contents in the isolated protein were determined by an amino acid analyzer. Fourier transform infrared (FTIR) spectroscopy and scanning electron microscopy (SEM) were used to analyze the extract’s functional groups and surface morphology. The extracted protein exhibited 51.08% *β*-sheet conformation. No adverse effects were observed in extract-treated cells, indicating their biocompatibility. The protein isolate showed an excellent antioxidative property. Besides this, an enhancement in the hBMSCs’ mineralization has been observed in the presence of treated protein isolates. Notably, osteogenic marker genes and proteins were effectively expressed in the treated cells. These results indicated that the *P. brevitarsis*-derived protein isolate can be used as a potential antioxidative biomaterial for bone tissue engineering applications.

## 1. Introduction

The formation of new bone tissue depends upon the osteogenic development of associated stem cells [1]. Human bone-marrow-derived mesenchymal stem cells (hBMSCs) have an exceptional ability to heal bone-related diseases [2]. The physiological niche of hBMSCs, including the tissue type and bioactive molecules, dramatically determines their osteogenic differentiation potential. Under pathological conditions such as bone cancer, osteoporosis and bone infections, the viability and differentiation of hBMSCs are markedly impaired, leading to delayed tissue healing and recovery [3]. Stem cell-based regenerative medicine has shown significant therapeutic promise. Regulating the stem cell differentiation potential can benefit existing tissue engineering practices remarkably. In this regard, bioactive compounds such as proteins and small molecules are crucial to cellular differentiation [4].

Insects have been considered a great alternative source for bioactive molecules. Insect-derived proteins can promote stem cell proliferation and exhibit antioxidative properties. *Aspongopus chinensis* (Chinese stinkbug)-derived nonpeptide small molecules have stimulated neural stem cell proliferation significantly [5]. Compounds derived from Bruchidius dorsalis (Seed beetle) have antioxidative properties that are comparable to the commercially available Trolox antioxidant [6]. *Protaetia brevitarsis seulensis* (white-spotted flower chafer)-derived protein hydrolysates have been used as medicinal material in many Asian countries for their antioxidative, anticancer, antiobesity, antidiabetic, and hepatoprotective effects [7,8,9,10]. Controlled feeding patterns have also modulated the bioactive substance’s synthesis in *P. brevitarsis* larvae for its potential exploitation [11]. The development of health-promoting functional materials from *P. brevitarsis* extracts have also been investigated [12]. However, the osteoinductive potential of *P. brevitarsis*-derived protein material has yet to be understood.

Herein, we have evaluated the osteoinductive and antioxidative potential of *P. brevitarsis*-derived protein isolate (PPI) in hBMSCs’ osteogenic differentiation. The morphological and chemical characterization of the extracted protein isolates revealed their biocompatible surface architecture, significant functional groups, peptide range, and amino acid composition. Furthermore, PPI also showed an excellent antioxidative property and greatly supported hBMSCs’ osteogenic differentiation. However, the osteogenic differentiation of hBMSCs was not significantly enhanced in the presence of PPI. Based on our findings, we propose that PPI is an efficient biologically active, biocompatible, and antioxidative complex protein material that holds the potential for the expansion and osteogenic differentiation of hBMSCs along with an antioxidative property.

## 2. Materials and Methods

### 2.1. Materials

*Protaetia brevitarsis* larvae were obtained from a local farm (Godae farm, Wonju, Korea). The hBMSCs were received from the Korean Cell Line Bank (KCLB, Seoul, Republic of Korea). Laemmli buffer (5×) and protein ladder were purchased from Dyne Bio Inc., Seongnam, Republic of Korea. Coomassie Brilliant Blue and SYBR Green Master mix were supplied by Bio-Rad Laboratories, USA. Dulbecco’s Modified Eagle’s Medium (DMEM), 10% fetal bovine serum (FBS), Dulbecco’s phosphate-buffered saline (DPBS), and antibiotics were purchased from Welgene Inc., Gyeongsan, Republic of Korea. Trypsin-ethylene diamine tetra acetic acid (Trypsin-EDTA) was provided by Gibco, Gaithersburg, MD, USA. Osteo-induction media, 2,7-dichlorofluorescein diacetate (DCF-DA), alizarin red staining (ARS), and alkaline phosphatase (ALP) staining kits were acquired from Sigma–Aldrich, USA. WST-1 dye was purchased from DoGenBio Co., Ltd., Seoul, Republic of Korea. TRIzol^®^ reagent, Acridine orange, and Ethidium bromide stains were purchased from Invitrogen, Thermo Fisher Scientific, Waltham, MA, USA. The cDNA synthesis kit was obtained from Invitrogen, Gaithersburg, Carlsbad, CA, USA. The gene primers were supplied by BIONEER^®^Inc., Daejeon, Republic of Korea.

### 2.2. Defatting and Protein Extraction

*Protaetia brevitarsis* larvae were fed with wheat bran and harvested after 90 days from their hatching. The larvae were kept at −80 °C and lyophilized. The lyophilized samples were ground with a pulverizer (RT-N08, Rong Tsong Precision Technology, Taichung, Taiwan). The lyophilized larvae were defatted, as reported previously, with a slight modification [13]. The lyophilized powder was dispersed five-fold in ethanol (99.5%) and stirred for 4 h with filtering and replacing the ethanol at 2-h intervals. Afterward, the defatted residue was dried at room temperature. The defatted powder was mixed with 15-fold deionized water and stirred for 30 min. The pH of the mixture was adjusted to pH 2 by adding 2.5 M HCl and was agitated for 60 min at RT. The mixture was centrifuged at 3200× *g* for 20 min, and the supernatant was collected. The pH of the supernatant was adjusted to pH 4.6 by adding 2.5 M NaOH, and the mixture was centrifuged at 3200× *g* for 20 min. The pellet was collected and lyophilized. For the cell culture experiments, 0.1, 0.5, 1, and 2% of PPI concentrations were used.

### 2.3. Characterization of the Isolate

#### 2.3.1. Determination of the Nutrient Composition

The amino acid composition of *Protaetia brevitarsis* larvae protein isolate (PPI) was analyzed using an Amino Acid Analyzer (HITACHI L-8900, Hitachi High-Technologies Corporation, Tokyo, Japan) with the ninhydrin method. The PPI’s Proximate composition, including crude protein, crude fat, and crude ash, was analyzed according to the Association of Analytical Communities (AOAC) methods [14]. The carbohydrate content was calculated using the following equation:

Carbohydrate content (%) = 100% − Crude protein (%) − Crude fat (%) − Crude ash (%).

#### 2.3.2. Molecular Weight Determination

The molecular weight distribution of PPI was analyzed using Sodium dodecyl sulphate-polyacrylamide gel electrophoresis (SDS-PAGE), as described previously [15]. The samples dissolved in 8 M urea were mixed with 5× sample buffer (Dynebio, Seongnam, Korea) containing 62.5 mM β-mercaptoethanol, 10% SDS, and 0.1% Bromophenol Blue. The mixture was heated at 95 °C for 4 min. The protein ladder markers (Dynebio) at the ranges of 12–160 kDa (4 μL) and 35–245 kDa (4 μL) and the samples (10 μL) were loaded and separated in 10% and 8% Tris-Glycine gels, respectively, with an electrophoresis apparatus (PowerPac™ Basic, Bio-Rad Laboratories, Hercules, CA, USA). The gel was stained with 0.05% Coomassie Brilliant Blue and destained using acetic acid: methanol: deionized water (2:5:5, *v*/*v*/*v*).

#### 2.3.3. Chemical Composition and Morphological Analysis

The functional groups present in the isolate were determined by FTIR analysis using the Perkin Elmer FTIR analyzer (Frontier, Perkin Elmer, UK) at the wavenumber range of 4000–1000 cm^−1^ with a resolution of 4 cm^−1^. The prominent peaks in the FTIR spectrum near the amide I and II regions (1540–1640 cm^−1^) were determined by deconvolution using Origin Pro 9.0 software. The area of the obtained peaks was measured using the Gaussian function. The area of the individual bands was summed up and divided by the total area to estimate the percentage of the *β*-conformation in PPI. The morphological features of the isolate were determined by high-resolution field emission-scanning electron microscopy (FE-SEM) (S-4800, Tokyo, Japan).

#### 2.3.4. DPPH Assay

The DPPH assay was used to determine the radical scavenging activity of the isolate. Briefly, different concentrations of the isolates (0.1, 0.5, 1, and 2%) were prepared in a 0.4-mM DPPH solution, incubated for 30 min in the dark at room temperature. Equivalent concentrations of the ascorbate solution were prepared for reference. The optical density (OD) was recorded at 517 nm for all the samples using a spectrophotometer (Infinite^®^ M Nano 200 Pro; TECAN, Zürich, Switzerland). The OD values were plotted to compare the DPPH scavenging activity of the protein isolate with an equivalent concentration of ascorbate.

### 2.4. Cell Culture

The hBMSCs were received from the Korean Cell Line Bank (KCLB, Seoul, Republic of Korea) and cultured as previously reported. Briefly, the cell culture was carried out using DMEM supplemented with 10% FBS and 1% antibiotics containing penicillin (10,000 units/mL), streptomycin (10,000 µg/mL), and amphotericin B (25 µg/mL) at 37 °C in a humidified atmosphere of 5% CO_2_ (Steri-Cycle 370 Incubator; Thermo Fisher Scientific, Waltham, MA, USA). After 70–80% confluency, the hBMSCs were treated with different concentrations of PPI for the desired periods. Passage 5 cells were used in this study. For the osteogenic induction, the cells were cultured in an osteogenic induction media containing DMEM supplemented with 50 µg/mL L-ascorbic acid, 10 mM β-glycerophosphate, and 100 nM dexamethasone.

### 2.5. Cell Viability Assay

The hBMSCs (1 × 10^4^ cells/100 µL media) were seeded onto the 96-well plate and incubated at 37 °C with 5% CO_2_ atmosphere for five days in the presence of 0.1, 0.5, 1, and 2% concentrations of PPI. Plates without treatment were considered as the control set. The cell viability was analyzed using a WST-1 assay (EZ-Cytox Cell Viability Assay Kit^®^). After a specific time interval, 10 µL of the WST-1 dye was added and further incubated for 2 h. The produced formazan was quantitated by measuring the absorbance at 450 nm (625 nm as a reference value). All the experiments were accomplished in triplicate, and data are presented as the mean ODs ± standard deviations. Statistical significance was considered at * *p* < 0.05.

### 2.6. Live-Dead Assay

For this, the hBMSCs (4 × 10^4^ cells/100 µL media) were cultured in a four-well plate at 37 °C with 5% CO_2_, followed by a 0.5% PPI treatment after 70–80% of confluency. The cells grown in DMEM alone were taken as the control. The cells were washed with 1× PBS, followed by treating them with 1 µL of acridine orange and ethidium bromide dye solution at a ratio of 1:1. The images were captured immediately on appropriate filter channels using the Leica Microsystems Suite X software (Leica Microsystems, Wetzlar, Germany) of the inverted fluorescence microscope (DMi8 Series, Leica Microsystems, Germany). The survivability of the PPI-treated cells was quantified using the live-dead fluorescence imaging after five days of incubation.

### 2.7. Cell Morphology

The effect of PPI on the morphology of hBMSCs was investigated using Giemsa staining and fluorescence microscopy. For Giemsa staining, the hBMSCs (4 × 10^4^ cells/1 mL media) were cultured onto a 24-well plate in the presence of 0.5% PPI for three days. The media without treatment and gelatin were considered as the negative and positive control. After a 80% confluency, the cell surface was washed twice with PBS and fixed with 3.7% PFA at room temperature. After that, the fixed cells were permeabilized with 100% methanol for 20 min, followed by incubation with Giemsa stain for 10 min. The excess stain was removed by washing with PBS, and images were captured at a 5× magnification under an inverted optical microscope (Zeiss Optical Microscope, White Plains, NY, USA).

The hBMSCs (2 × 104 cells/500 µL media) were cultured in gelatin-coated cover slips and treated with 0.5% PPI for three days for fluorescence microscopy. The media without the PPI was taken as the control. The staining of cells was performed as described earlier, with some modifications [16,17]. Briefly, the cells were washed with PBS and fixed with 3.7% paraformaldehyde (PFA) for 15 min at room temperature, followed by the addition of 0.1% Triton X-100 to permeabilize the cells for 10 min at room temperature. Next, the cells were blocked by 1% bovine serum albumin for 1 h. After blocking, the cells were incubated with 2–3 drops of Alexa Fluor 488 F-actin probe (ex/em = 488/518) and vinculin antibody (1:200 dilution) to visualize the actin skeleton and vinculin protein. The nucleus was counterstained by 4,6-diamino-2-phenylindole dihydrochloride (DAPI) solution for 2 min in the dark. The stained cells were finally mounted and visualized with an inverted fluorescence microscope (Leica Microsystems, Wetzlar, Germany).

### 2.8. Assessment of Reactive Oxygen Species (ROS) Scavenging Property of PPI

The H_2_O_2_-induced oxidative stress in hBMSCs was assessed in the presence of 0.1, 0.5, and 1% of PPI by observing the formation of free radical species using DCF-DA as reported previously, with a slight modification [18]. Briefly, cells were cultured at a density of 2 × 10^4^ in the presence of 0.1, 0.5, and 1% PPI. Next, the PPI-treated cells and the positive control were incubated with 200-µM H_2_O_2_ for 20 min at 37 °C in the CO_2_ incubator. Cells without an H_2_O_2_ treatment were taken as the negative control. All cells were then fixed and permeabilized with 4% paraformaldehyde and 0.1% Triton-X 100, respectively, and incubated with 20 µM DCF-DA for 30 min. After that, the cells were washed with PBS, and the nucleus was counterstained with DAPI for 30 s. The DCF-DA fluorescence intensity was checked using a fluorescence microscope (ex/em = 485/538). The respective intensities of the DCF-DA were measured using ImageJ software (ImageJ v1.8, NIH Lab., Bethesda, MD, USA, https://imagej.nih.gov/) for the quantitative analysis of the formation of intracellular ROS.

### 2.9. In Vitro Osteogenic Differentiation Study

The effect of PPI on the mineralization of hBMSCs was evaluated by ARS staining after seven and 14 days of treatment. The hBMSCs (4 × 10^4^ cells/1 mL media) were cultured onto a 24-well plate in the presence of 0.1, 0.5, 1, and 2% PPI for seven and 14 days. The used media were replaced with fresh media every three days. After a specific interval, the cells were rinsed twice with PBS and fixed with 1 mL of 70% ice-cold ethanol for 15 min at room temperature. Next, the fixed cells were stained with 500 µL of 40 mM ARS (pH 4.2) stain for 10 min, followed by washing with deionized water to remove the excess stain. The mineralized nodule formation was captured by using an inverted optical microscope. After that, the stained plates were treated with 500 µL of destaining solution (10% cetyl pyridinium chloride and 10 mM of sodium phosphate). The absorbance of the solution was taken at 562 nm using a spectrophotometer. All the samples were prepared in triplicate, and data are presented as mean ODs ± standard deviations. Statistical significance was considered at * *p* < 0.05.

For the ALP activity, the hBMSCs were cultured for seven and 14 days with 0.5% PPI and stained by the Leukocyte Alkaline Phosphatase Kit according to the manufacturer’s protocol. Briefly, the treated cells were washed with PBS and fixed with the fixative solution for 1 min. After this, the cells were rinsed with distilled water and stained with the staining solution for 30 min. The stained cells were rinsed with distilled water and visualized with an inverted optical microscope with a magnification of 10×.

### 2.10. RNA Isolation and Real-Time PCR (qRT-PCR) Analysis

The expression of the osteogenic-related gene in the 0.5% PPI and control was evaluated by qRT-PCR technique. Briefly, the cells (4 × 10^4^ cells/100 µL media) were cultured in a 24-well plate in the osteogenic induction media for seven and 14 days, followed by the extraction of RNA by TRIzol^®^ reagent (Thermo Fisher Scientific, USA), according to the manufacturer’s instructions. The purity and concentration of the extracted RNA were evaluated by a spectrophotometer. The cDNA was synthesized from 2 µg of RNA by using reverse transcriptase and SYBR Green Master mix. The mRNA expression was quantified with a Bio-Rad Real-Time PCR (CFX96TM Maestro Real-Time System, Bio-Rad, USA). The reaction condition included 43 cycles of denaturation for 15 s at 95 °C and a 1 min amplification at 60 °C. All the experiments were performed in triplicate and normalized to the housekeeping gene *β*-actin. The relative mRNA expression from hBMSCs in the presence of PPI and control was compared in a histogram. All the samples were prepared in triplicate during the experiments. The specific gene primers used for the qRT-PCR analysis are listed in Table 1.

### 2.11. Protein Marker Expression Analysis

The expression of the osteogenic marker proteins was studied through fluorescence imaging. The hBMSCs (2 × 10^4^ cells/ 500 µL media) were cultured in 60-mm bottom well plates and treated with 0.5% PPI for seven and 14 days. The media without the PPI was taken as the control. The staining of the cells was performed by washing with PBS. Next, the cells were fixed with 3.7% paraformaldehyde (PFA) for 15 min at RT. After that, the cells were permeabilized by the addition of 0.1% Triton X-100 for 10 min at RT. Then, the cells were rinsed twice with PBS, blocked by 1% BSA, and incubated with 250 µL of mouse monoclonal antibodies against Runx2, ALP, and OPN. The specific antibody dilutions are listed in Table S1. The nucleus was counterstained with 20 µL of 1 mg/mL DAPI solution for 2 min in the dark. The stained cells were rinsed and covered with a mounting medium and a glass coverslip. The fluorescence images were taken with a fluorescence microscope at a magnification of 40×. The fluorescence intensity of the images was quantified using ImageJ software (ImageJ v1.8, NIH Lab., Bethesda, MD, USA, https://imagej.nih.gov/).

### 2.12. Statistical Analysis

Statistical analysis was performed using Origin Pro 9.0 software. Statistical significance between the control and treatment groups was determined using a one-way ANOVA. All the data are presented as mean ± SDs. Differences were considered significant at * *p* < 0.05.

## 3. Results and Discussion

### 3.1. Nutritional Composition

**Figure 1:**
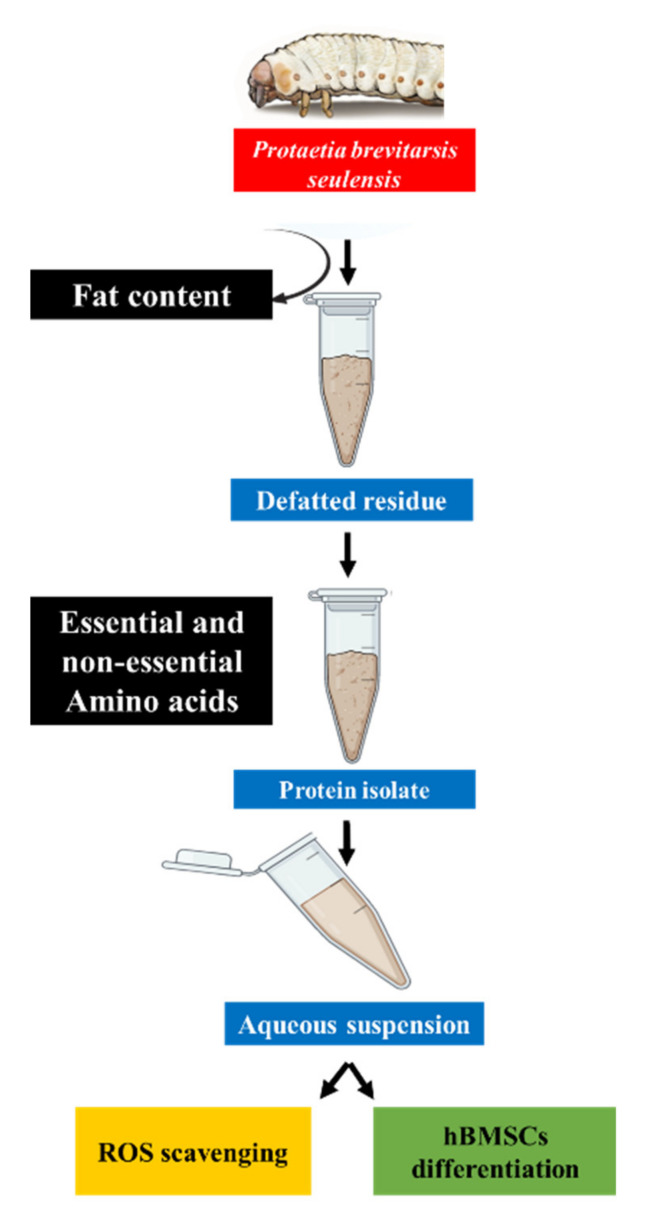
Schematic illustration of protein extraction from *Protaetia brevitarsis seulensis*.

Table 2 shows the nutritional composition of the PPI. The extraction yield demonstrated a significant amount of protein content (~77%) compared to carbohydrate (~17%), crude fat (~0.52%), and crude ash (~4%). Our extracted protein yield is consistent with the previously reported extraction process yields of up to ~73% implemented in PPI isolation [10]. Protein supplements efficient in modulating MSCs’ differentiation are of significant clinical and commercial interest [19]. The protein yield is crucial in determining the PPI’s amino acid profile, which serves as a precursor for hBMSC osteogenesis [20]. The PPI isolation process is represented in Figure 1.

Amino acid supplementation greatly determines the potential of osteogenic differentiation of hBMSCs under culture conditions [21]. Hence, we determined the amino acid composition of PPI, and the values are listed in Table 3**.** The total amount of amino acid of the isolate was found to be 75.40 g/100 g of the sample. The data exhibited that the protein extract was ~36% essential and ~38% nonessential amino acids. Leucine is the highest amount of essential amino acids (5.92 g), followed by lysine and tyrosine (5.31 and 5.24 g), per 100 g of PPI, respectively. Among the nonessential amino acids, glutamic acid (9.68 g) and aspartic acid (7.48 g), per 100 g of PPI, were relatively higher in concentration. The abundance of glutamic acid in our extracted PPI (9.15 g/100 g sample) is expected to be a crucial factor in promoting the hBMSCs’ differentiation. Glutamine has long been recognized as a vital amino acid for stem cell differentiation into osteoblasts [22] and bone homeostasis [23].

Additionally, the abundance of aspartic acid content in PPI (8.44 g/100 g sample) also possibly facilitated the cultured cells’ osteogenic differentiation. Aspartic acid has been shown to promote osteogenic differentiation more than glutamic acid when conjugated as a template peptide on nanofiber for the osteogenic differentiation of hMSCs [24]. Aromatic amino acids in PPI might also trigger osteogenic differentiation upon entering the cells through the multiple amino acid receptors in hBMSCs [25,26].

### 3.2. Characterization of the PPI

**Figure 2:**
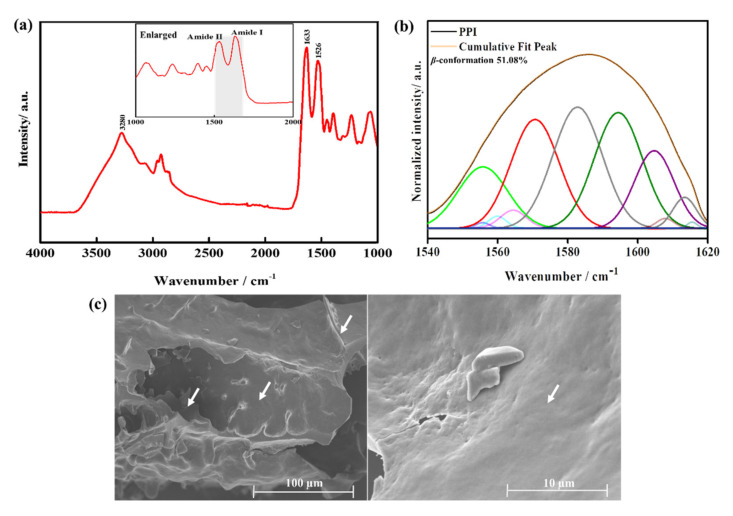
Chemical characterization of PPI. (**a**) The FTIR spectrum of the PPI extracted from the insect, (**b**) curve-fitted amide I and II region (1540–1620 cm^−1^), and (**c**) FE-SEM morphology of freeze-dried PPI; the white arrows (left to right) indicate the rough, appendicular ridges, and the smooth surfaces present throughout indicate the protein isolate, respectively.

FTIR is one of the useful methods for the determination of a protein structure [27]. Figure 2a shows the FTIR spectrum of the PPI, showing typical protein absorption peaks in the range of 4000–1000 cm^−1^ [28]. The appearance of the absorption peaks at 1633 and 1529 cm^−1^ indicates the –C=O (carbonyl) and –N-H (amide II) groups. Antiparallel *β*-sheet conformation has been reported to exhibit a strong band near 1630 cm^−1^ [29,30,31]. It was evident from the FTIR spectrum that the PPI primarily consisted of protein isolates of antiparallel *β*-sheet conformation. The FTIR peaks, along with the abundance of valine, isoleucine, and threonine, correlate with the possible involvement of β-sheet conformations in the PPI. The percentage of *β*-conformation in the PPI was estimated to be around 51.08% by analysis of the deconvoluted FTIR spectra in the region near amide I and amide II (1540–1640 cm^−1^) as shown in Figure 2b. Our results show the presence of resolvable peaks near the reported range of 1600–1700 cm^−1^ [32]. The *β*-sheet rich polypeptides can mimic the extracellular matrices for stem cell culture [33]. The FTIR absorption peak at 3280 cm^-1^ indicates the existence of –OH (hydroxyl) or –NH_2_ (amine) groups of the protein [34]. The surface topographical features, including the surface pattern and roughness of the material, play a significant role in cell survivability and osteogenic differentiation.

The FE-SEM analysis of the surface morphology of the freeze-dried PPI and the micrographs are shown in Figure 2c and Figure S1. The PPI exhibited a combination of a rough and smooth layer of flakes in the morphology, suggesting a crystallinity in their structure. Additionally, appendicular structures are occasionally seen on the surface of the PPI. However, the surface morphology is greatly affected by the extraction process and its conditions. The zeta potential measurement was performed to measure the surface potentials of the PPI in water. The colloidal solution exhibited a zeta potential value of 23.2 mV ± 3.72, indicating that the PPI suspension was electrically stabilized.

### 3.3. Molecular Weight Analysis

**Figure 3:**
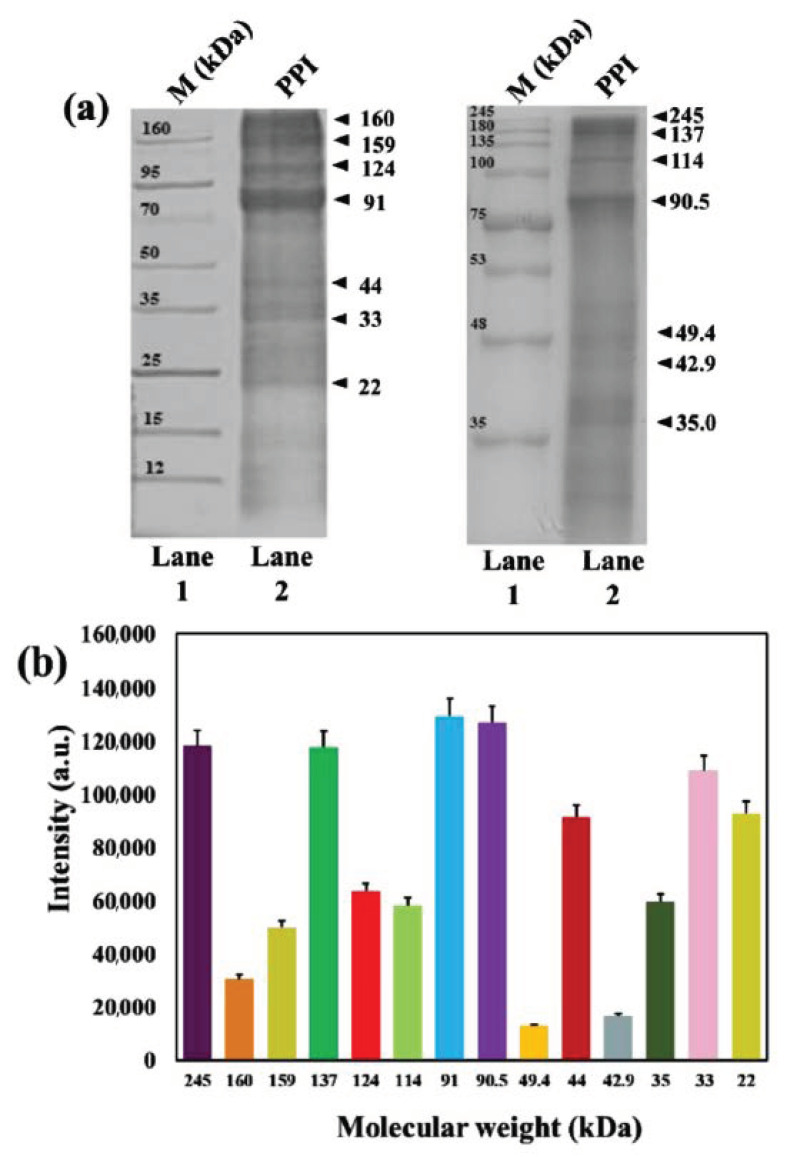
Determination of the molecular weight of PPI. (**a**) SDS-PAGE analysis of total PPI, and (**b**) SDS-PAGE band intensity profile of total PPI.

The SDS-PAGE analysis was performed to know the molecular weights of the protein content of the PPI, and the results are shown in Figure 3a. Lane 1 indicates the protein ladder, while lane 2 shows the PPI protein composition, including fourteen prominent protein bands and a smear of several other faint bands. The protein contents were determined from their respective intensities, and the band intensity profile is given in Figure 3b. The protein fraction of 91 kDa exhibited the highest band intensity, followed by 90.5, 137, 245, 33, 22, 44, 124, 35, 114, 159, 160, 42.9, and 49.4 kDa molecular weight values, suggesting that the PPI solution consists primarily of a wide range of proteins. Our result is consistent with the previously reported protein fractions of extracted protein isolate from *P. brevitarsis* [35].

### 3.4. Antioxidant Activity of the PPI

**Figure 4:**
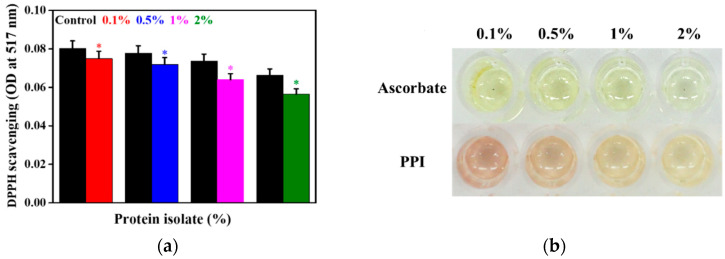
Measurement of the antioxidant property. (**a**) DPPH scavenging activity of PPI, with reference to an equivalent concentration of ascorbate. (**b**) Digital photographs of the plates incubated with different concentrations of PPI. Data are the mean OD ± SD of triplicated experiments; statistical significance at * *p* < 0.001.

The antioxidant potential of PPI was determined using a DPPH assay in the presence of different concentrations of the PPI, and the results are presented in Figure 4a. We observed a decrease in the absorbance at 517 nm under PPI-treated conditions when compared to the control, showing their scavenging property. This property is highly affected by the PPI concentrations, and among them (0.1, 0.5, 1, and 2%), 2% demonstrated a better scavenging potential. The photographs of the DPPH reduction in the presence of different concentrations of PPI and ascorbate are shown in Figure 4b. A more transparent solution was observed as the concentrations of PPI increased, showing their better scavenging potential. At higher concentrations (2%), the PPI solution’s transparency was approximately similar to the ascorbate. The concentration-dependent scavenging potential of PPI is attributed to numerous factors, including the amino acid composition [36], the presence of different functional groups in their structure, and lower molecular weight peptide fractions in the PPI [37,38]. Significantly, low molecular weight peptides (≤3 kDa) have been reported to show an enhanced antioxidant activity when compared to larger peptides [39].

### 3.5. Cell Viability and Morphology

**Figure 5:**
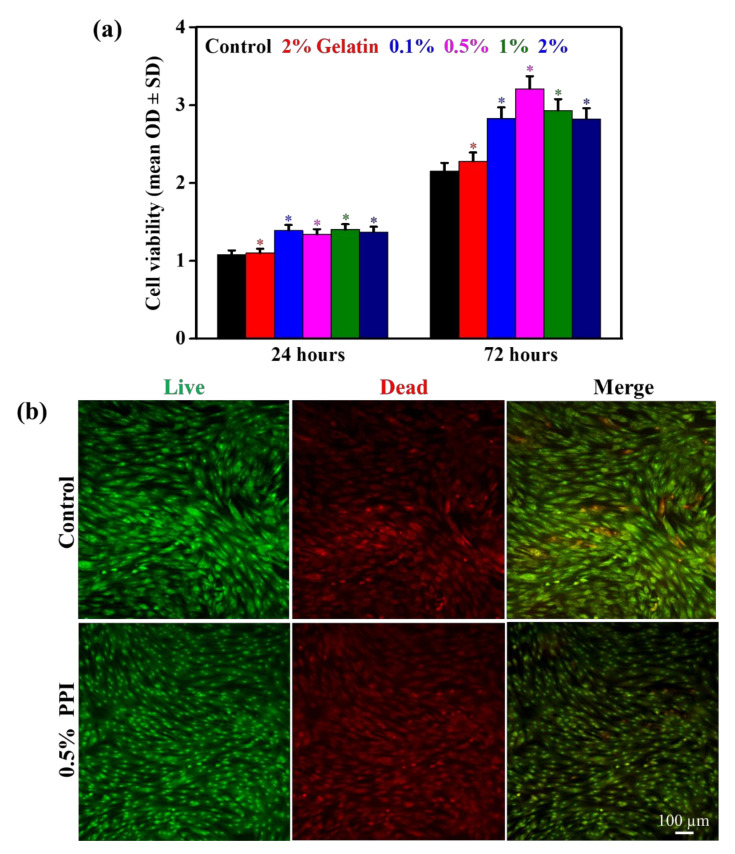
In vitro cytotoxicity evaluation of PPI on hBMSCs at indicated time intervals. (**a**) WST-1 assay of PPI-treated hBMSCs. (**b**) Live/dead assay of 0.5% PPI-treated hBMSCs after a three-day interval.

Biocompatibility is an essential requirement of any therapeutic agent. We investigated the possible cytotoxicity of PPI before determining their osteoinductive potential. Cytotoxicity of the PPI on the hBMSCs was monitored through a WST-1 assay, and the results are presented in Figure 5a. It was interesting to see that the PPI concentrations altered the cell viability significantly, and 0.5% PPI-treated media exhibited a higher cell viability after 72 h of treatment, indicating a suitable dose for cellular activity. The biocompatibility of the PPI was compared to gelatin, which is an excessively used protein in tissue regeneration. Our results indicated an increased percentage of viable cells up to a concentration of 0.5% PPI. However, the cellular viability was reduced at higher concentrations of PPI, which was attributed to the presence of a higher amount of suspended nutrient fraction in the PPI restricting the proliferation of hBMSCs. This is evident by the composition analysis of PPI, which shows a notable percentage of fat, ash, and carbohydrate (Table 2). Besides, the flaky and crystalline nature of the PPI, as shown in Figure 2c, might also have undesirable topological effects on hBMSCs’ growth. Figure 5b shows the live-dead assay for hBMSCs in the presence of 0.5% PPI and control after three days of treatment. The number of cells that adhered to the substrate was estimated by counting the number of the nucleus. The cell death was significantly lower in the PPI-treated media than in the control, further showing their improved biocompatibility.

**Figure 6:**
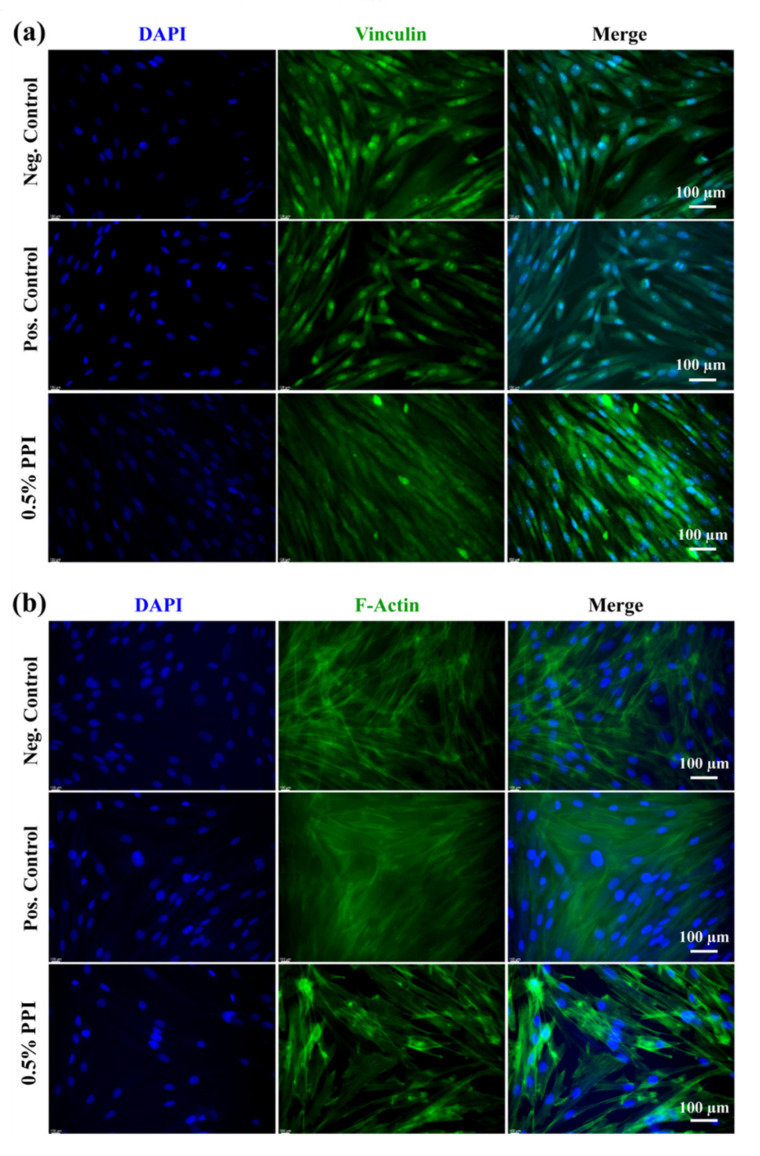
Morphology of PPI-treated hBMSCs after three days of incubation. Fluorescence images of (**a**) vinculin and (**b**) actin.

The morphology of hBMSCs in the presence of PPI was monitored using Giemsa staining after three days of incubation, and the images are shown in Figure S2. The cells without PPI and with gelatin were considered as a negative and positive control, respectively. A similar colony formation pattern was observed in the PPI-treated media and in the control, showing their biocompatibility. The expression of F-actin and vinculin was assessed through immunofluorescence staining after three days of incubation, and the results are shown in Figure 6. The media without PPI were taken as the control. The actin and nuclear morphologies of hBMSCs were comparable in the PPI-treated media and the control cells, showing no observable cytoskeletal or nuclear change in the PPI-treated cells. Interestingly, the localization of vinculin proteins appeared to be normal in both the control and PPI-treated cells, indicating the PPI’s biocompatible nature. We also observed that the extracted PPI was highly biocompatible, as evidenced by the increased viability, reduced cell death, unaltered colony formation pattern, and the absence of apparent cytoskeletal/nuclear morphological damages in hBMSCs upon PPI treatment. Nevertheless, both health-promoting [40,41] and safety concerns [42] regarding *P. brevitarsis* consumption have been reported. The biocompatibility is also greatly determined by the surface morphology of the biomaterial [43].

### 3.6. Intracellular Reactive Oxygen Species (ROS) Scavenging Activity of PPI

**Figure 7:**
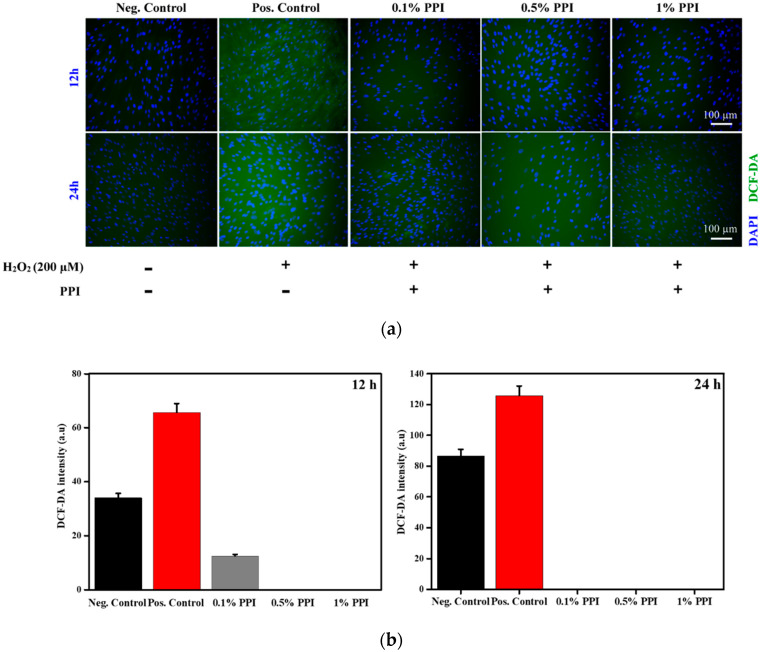
Assessment of the ROS scavenging potential of PPI under H_2_O_2_-induced oxidative stress. (**a**) DCF-DA staining at indicated time intervals, and (**b**) the respective image-based ROS measurement.

To investigate the antioxidant property of hBMSCs in the presence of PPI, we have performed H_2_O_2_-induced oxidative stress by H_2_DCF-DA staining, as shown in Figure 7a,b. A basal level of ROS production was observed in the negative control, indicating a physiological ROS production. Under physiological conditions, ROS formation occurs via the partial reduction of molecular oxygen [44]. An increased H_2_O_2_-induced ROS production was observed in the positive control cells. However, the H_2_O_2_-induced ROS production was dramatically reduced in the PPI-treated (0.1, 0.5, and 1%) cells after 12 and 24 h of PPI treatment, confirming the protein isolate’s antioxidative potential. We chose to exclude 2% PPI from our study henceforth, since the 2% PPI extract showed a slight decrease in cell viability. Our result is in accordance with the previously reported antioxidative property of *P. brevitarsis* protein extracts reported under varying extraction processes [45].

### 3.7. Mineral Induction in the Presence of PPI

**Figure 8:**
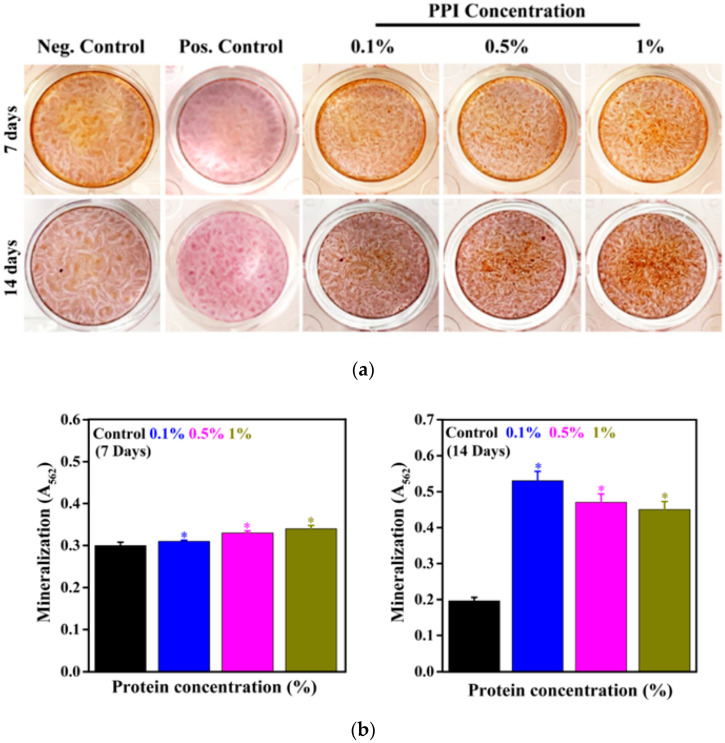
Evaluation of the in vitro mineralization of hBMSCs in the presence of PPI. (**a**) ARS staining of PPI-treated hBMSCs after seven and 14 days of incubation. (**b**) Mineralization potential of hBMSCs at indicated time intervals. Data are the mean ± SD of triplicated experiments; statistical significance at * *p* < 0.05.

The mineralization potential of hBMSCs in the presence of PPI was determined using ARS staining after seven and 14 days of treatment, and the results are shown in Figure 8a. An increase in the nodule formation was observed in PPI-treated cells when compared to the control after seven days of treatment, indicating their mineralization potential. This potential got further increased after 14 days of treatment. It was interesting to note that the mineralization potential was intensely affected by PPI concentrations in the media. Among these (0.1, 0.5, and 1%), 0.5% and 1% PPI-treated cells exhibited a higher and comparable mineral deposition potential. The quantitative values of the mineralized nodule are given in Figure 8b. The higher quantitative values confirm the superior mineralization potential of PPI. Similar results have been obtained with whey protein treatment for bone-forming cells’ osteogenic differentiation [46]. The ALP activity of the PPI-treated hBMSCs was evaluated after seven and 14 days of treatment, and the results are shown in Figure S3. An increased red coloration was observed in PPI-treated groups when compared to the control after seven days of treatment, indicating ALP expression. Thus, our results indicate PPI’s mineralization property and that they can be a possible alternative to animal-derived protein isolates for bone tissue engineering applications.

### 3.8. Gene and Protein Marker Expression

**Figure 9:**
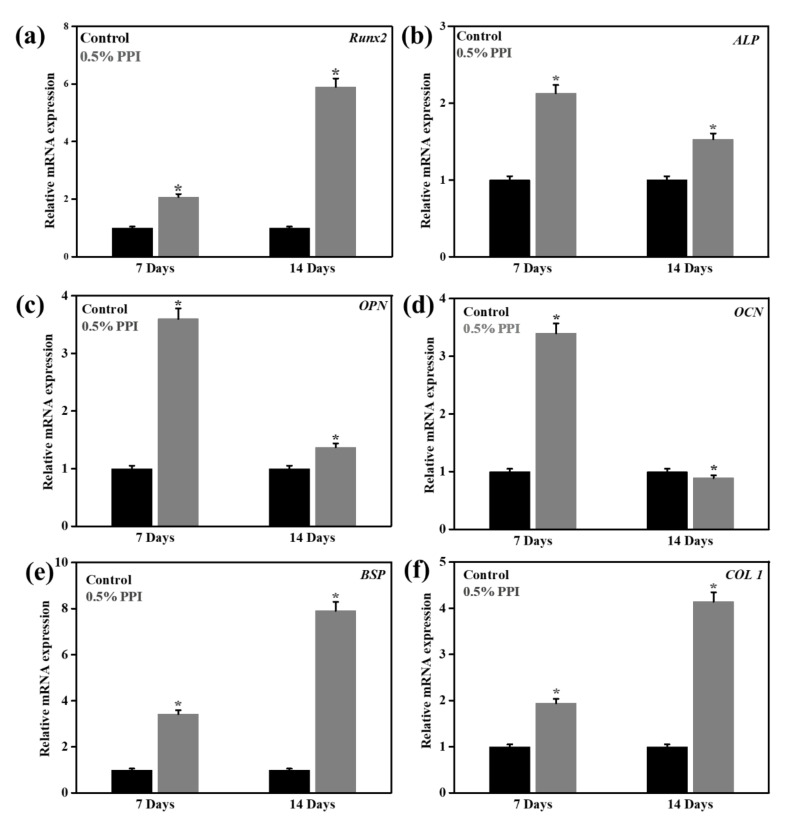
Real-time polymerase chain reaction (qRT-PCR) analysis of PPI-treated hBMSCs after indicated time intervals for, (**a**) *Runx2*, (**b**) *ALP*, (**c**) *OPN*, (**d**) *OCN*, (**e**) *BSP*, and (**f**) *COL1*. Data are the mean ± SD of triplicated experiments; statistical significance at * *p* < 0.05.

**Figure 10:**
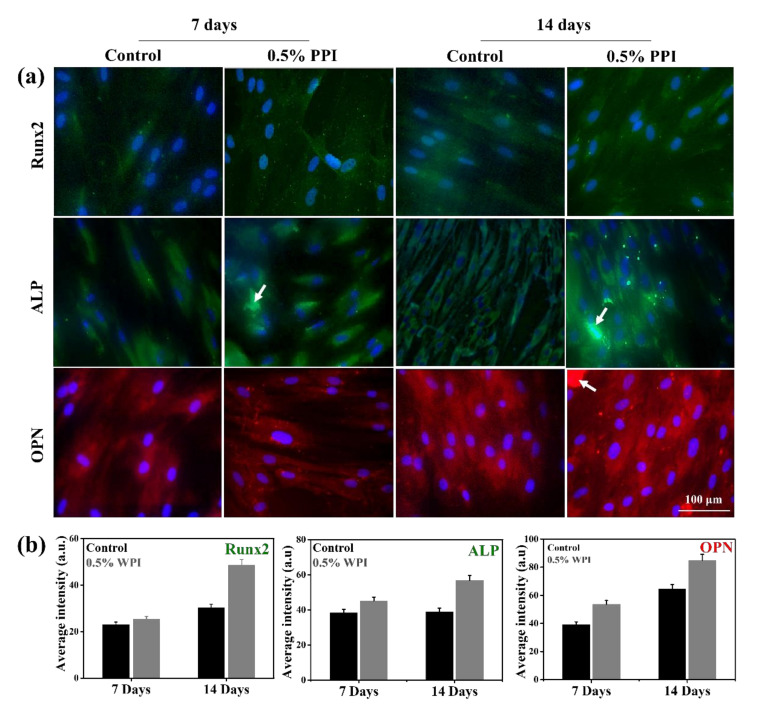
Osteoblast-specific protein markers’ expression of PPI-treated hBMSCs after seven and 14 days of treatment. (**a**) Fluorescence microscopy images of the respective protein markers (scale bar: 100 µm), and (**b**) corresponding fluorescence intensities. The white arrow indicates the presence of PPI on the surface of hBMSCs.

The expression of osteogenic marker genes (*Runx2*, *ALP*, *OPN*, *BSP*, and *COL1*) from hBMSCs in the presence of 0.5% PPI and control after seven and 14 days of treatment are shown in Figure 9. *Runx2*, *BSP*, and *COL1* expression were higher at up to 14 days of treatment. Conversely, the expression of *ALP* and *OPN* decreased after 14 days of PPI treatment. The osteogenic marker genes are dynamically expressed during osteogenesis and often altered through protein supplementations [47,48]. Our results indicated that PPI was a potential modulator of the osteogenic gene expression, as compared to the well-established expression profile of osteogenic gene markers [49]. *Runx2* is essential for the hBMSCs’ differentiation into the osteoblastic lineage, and its expression at the immature osteoblastic stage is reported to be the highest [50]. As we have studied the differentiation of the hBMSCs at up to 14 days of in vitro culture, our result indicates the probable onset of osteoblastic commitment. *ALP* is another crucial gene marker in the osteogenic development of hBMSCs [51]. Our result indicated a higher expression of *ALP* at seven days than at 14 days of PPI treatment. The *ALP* expression profile in our treated cells was consistent with the reported ALP expression during the early stage of osteoblast differentiation. Besides, the expression of OCN and OPN was found to be lower in the cells treated with PPI for 14 days than in those treated with PPI for seven days. OPN is recognized as promoting osteogenesis at the osteoblast and osteoclast stages [52]; however, OPN-deficient mice have also been reported to not show any major defect in bone mineralization [53,54]. The expressions of *BSP* and *COL1* were also found to be highly expressed after 14 days of PPI treatment, indicating PPI as a potential osteoinductive protein isolate [55,56]. The gene expressions were also confirmed with the respective protein expressions, as shown in Figure 10. The proteins were found to be expressed equally in the control and the treated cells, and no significant differences were observed in their fluorescence intensities. However, an enhancement was observed in the ALP and OPN expression intensities, which is attributed to the cumulative intensity of the deposited PPI. The expression profile of the marker genes and proteins indicates that PPI promotes early osteogenesis and does not significantly induce late osteogenesis.

## 4. Conclusions

We have extracted ~77% protein mass from *P. brevitarsis* and investigated its osteoinductive and antioxidative properties on hBMSCs. The nutritional composition showed the abundance of essential amino acids in the isolated protein. The presence of a wide range of peptides in the isolate, as indicated by their molecular weights, reflects their possible combined effect in stem cell fate determination. The extracted protein isolate (PPI) most likely exhibited a *β*- sheet confirmation (~51.08%). An enhanced cell viability was observed in PPI-treated hBMSCs when compared to the control, showing their excellent biocompatibility. The PPI also exhibited an enhanced antioxidant potential when compared to the control. Furthermore, an increase in the mineralization and osteogenic biomarkers’ expression indicated PPI’s potential as an excellent osteomodulatory agent. Therefore, *P. brevitarsis*-derived protein isolate is a functional protein material for reducing oxidative stress and could be used as an ideal material for bone tissue engineering.

## Figures and Tables

**Table 1:** Specified gene primer sequences used in the qRT-PCR analysis.

Genes	*GenBank* Accession No.	Sequences (5′ to 3′)
*β-actin*	NM_031144	ACCCGCGAGTACAACCTTCT CTTCTGACCCATACCCACCA
*Runx2*	NM_001146038	CGCACGACAACCGCACCAT CAGCACGGAGCACAGGAAGTT
*BSP*	L09555	AACTTTTATGTCCCCCGTTGA TGGACTGGAAACCGTTTCAGA
*ALP*	NM_007431	CCAACTCTTTTGTGCCAGAGA GGCTACATTGGTGTTGAGCTTTT
*OPN*	J04765	TGAAACGAGTCAGCTGGATG TGAAATTCATGGCTGTGGAA
*COL1*	NM007742	GCTCCTCTTAGGGGCCACT CCACGTCTCACCATTGGGG

**Abbreviations:***β*-actin; Actin beta, *Runx2*; Runt-related transcription factor-x2, *BSP*; Bone sialoprotein, *ALP*; Alkaline phosphatase, *OPN*; Osteopontin, *OCN*; Osteocalcin, and *COL1*; Collagen type-1.

**Table 2:** Composition (g/100 g) of the larva of *Protaetia brevitarsis seulensis* protein isolate (mean ± S.D., n = 3). The different letters in each column show a significant difference (* *p* < 0.05) between means. Carbohydrate (%): 100 – crude protein – crude fat – crude ash.

Sample	Crude Protein (%, DM)	Crude Fat (%, DM)	Crude Ash (%, DM)	Carbohydrate * (%, DM)
*P. brevitarsis* protein isolate	77.52 ± 0.31 ^a^	0.52 ± 0.14 ^c^	4.07 ± 0.35 ^b^	17.89

**Table 3:** Amino acid composition (g/100 g sample) for the protein isolate of *Protaetia brevitarsis seulensis*.

Essential Amino Acid	Contents (g/100 g Sample)	Non-Essential Amino Acid	Contents (g/100g Sample)
Isoleucine	3.76	Aspartic acid	7.48
Leucine	5.92	Serine	3.39
Lysine	5.31	Glutamic acid	9.68
Methionine	1.47	Proline	5.44
Phenylalanine	3.93	Glycine	3.22
Tyrosine	5.24	Alanine	3.61
Threonine	3.63	Cysteine	1.54
Valine	4.12	Arginine	4.33
Histidine	2.26	Non-essential A.A	38.69
Tryptophan	1.07		
Essential A.A	36.71		

## Supplementary Materials

The following are available online, Figure S1: Representative FE-SEM morphologies of *P. brevitarsis* protein isolate (PPI), Figure S2: Giemsa staining of PPI-treated hBMSCs after 3 days of incubation, Figure S3: ALP activity of hBMSCs after 7 and 14 days of incubation with *P. brevitarsis* protein isolate (PPI), Table S1: List of antibodies used for immunofluorescense staining.
