# Supplementary material for: Protaetia brevitarsis seulensis Derived Protein Isolate with Enhanced Osteomodulatory and Antioxidative Property
Source: Molecules. 2020 Dec 21;25(24):6056. doi: 10.3390/molecules25246056 (PMC7767527; doi:10.3390/molecules25246056)
Supplement: Supplementary file 1 [file molecules-25-06056-s001.pdf]

Protaetia brevitarsis seulensis Derived Protein Isolate with Enhanced Osteomodulatory and Antioxidative Property

Keya Ganguly[1], Min-Soo Jeong[2], Sayan Deb Dutta[1], Dinesh K. Patel[1], Seong-Jun Cho[2*], and Ki-Taek Lim[1*]

[1]Department of Biosystems Engineering, College of Agriculture and Life Sciences, Kangwon National University, Chuncheon-24341, Republic of Korea.

[2]Department of Food Science and Biotechnology, College of Agriculture and Life Sciences, Kangwon National University, Chuncheon-24341, Republic of Korea.

Supplementary Figures

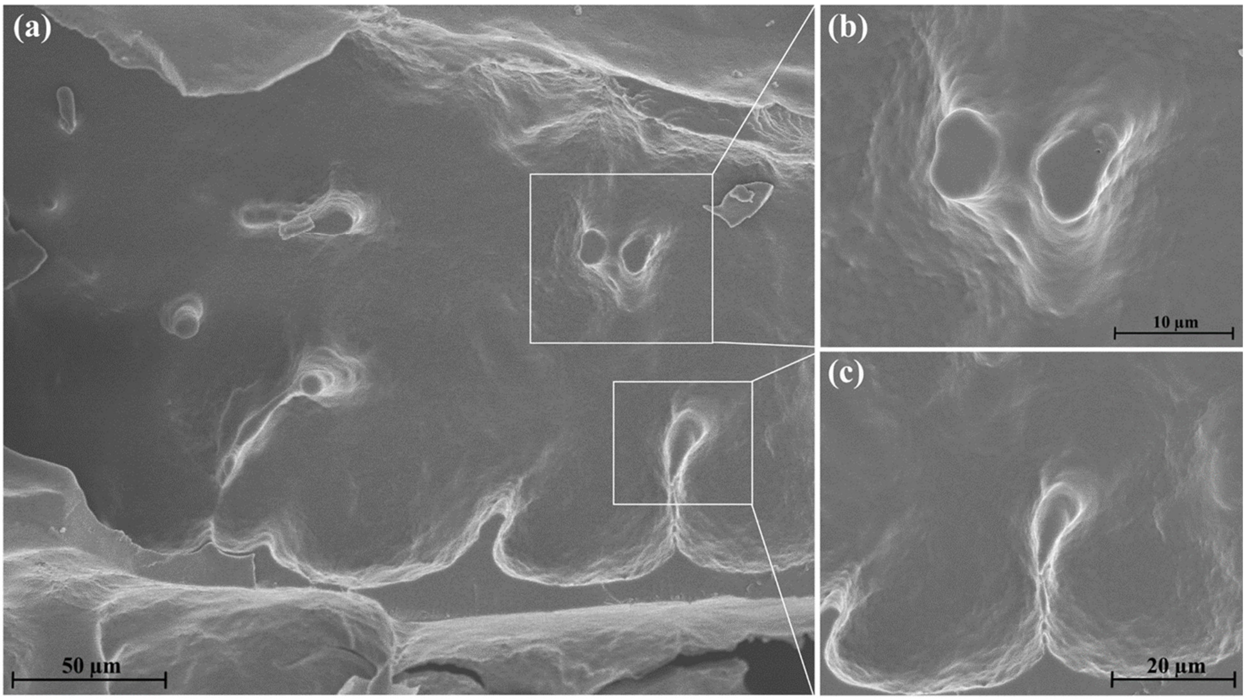

Figure S1. Representative FE-SEM morphologies of *P. brevitarsis* protein isolate (PPI).

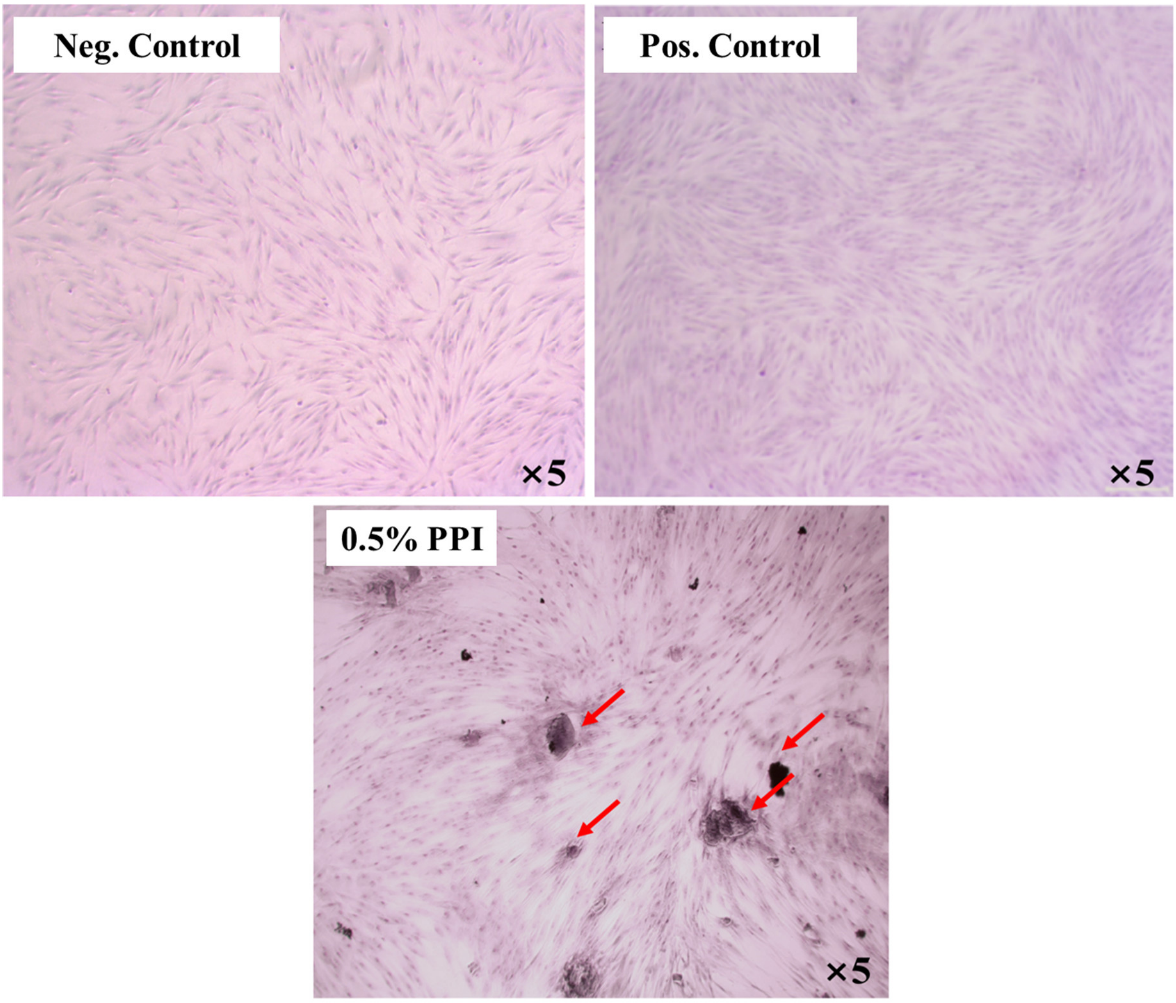

Figure S2. Giemsa staining of PPI-treated hBMSCs after 3 days of incubation. The red arrows indicate the *P. brevitarsis* protein isolates (PPI).

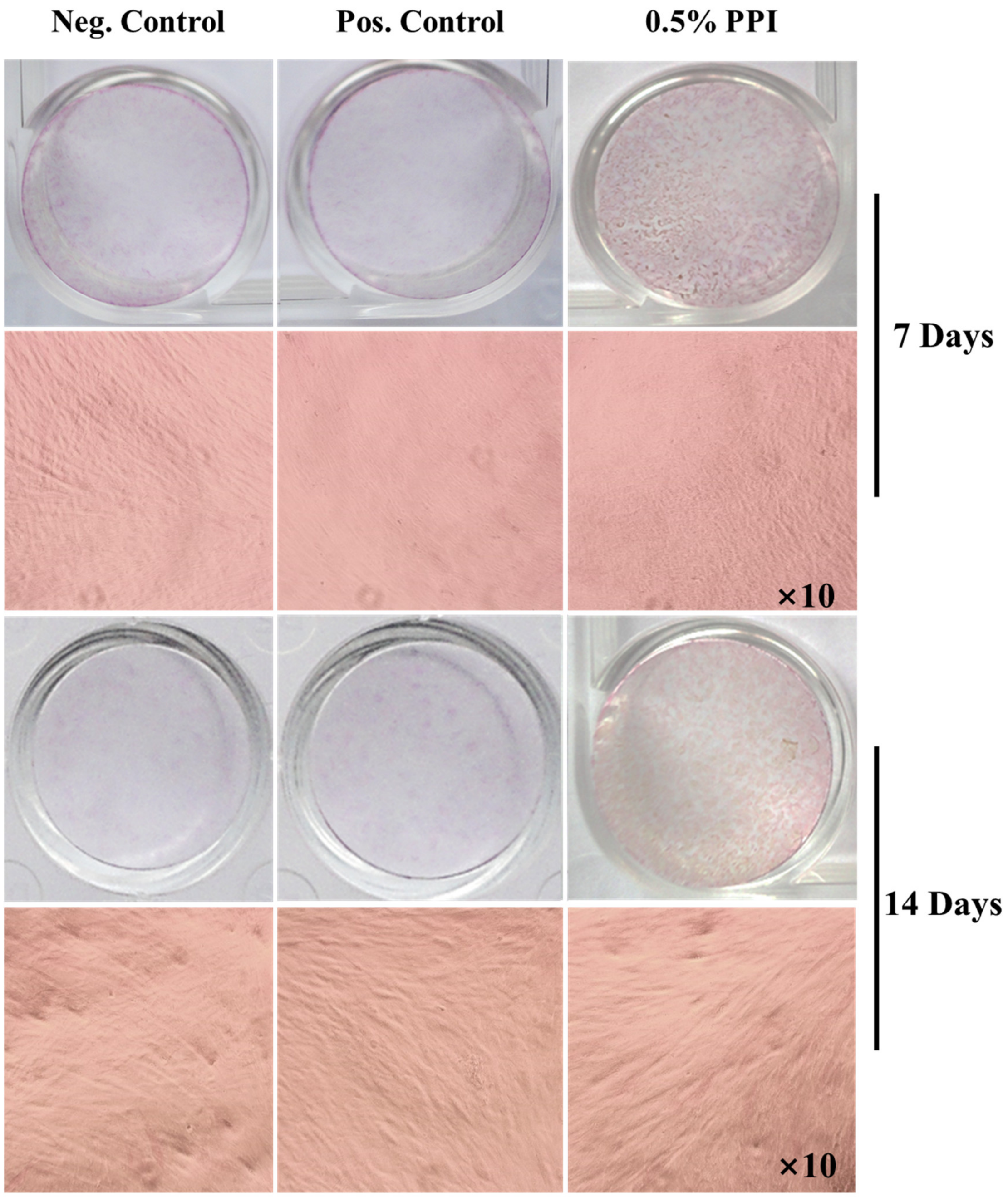

Figure S3. ALP activity of hBMSCs after 7 and 14 days of incubation with *P. brevitarsis* protein isolates (PPI).

Table S1. List of antibodies used for immunofluorescence staining.

Antibody	Dilution	Dye	Company
Runx2	1:250	AF-488	Santa Cruz Biotechnology, USA
ALP	1:250	AF-488	Santa Cruz Biotechnology, USA
OPN	1:250	AF-594	Santa Cruz Biotechnology, USA
